# Capacitive and Inductive Characteristics of Volatile Perovskite Resistive Switching Devices with Analog Memory

**DOI:** 10.1021/acs.jpclett.4c00945

**Published:** 2024-06-13

**Authors:** Cedric Gonzales, Agustín Bou, Antonio Guerrero, Juan Bisquert

**Affiliations:** † Institute of Advanced Materials (INAM), Universitat Jaume I, 12006 Castelló, Spain; ‡ Leibniz-Institute for Solid State and Materials Research Dresden, Helmholtzstraße 20, 01069 Dresden, Germany; § Instituto de Tecnología Química (Universitat Politècnica de València-Agencia Estatal Consejo Superior de Investigaciones Científicas), Av. dels Tarongers, 46022, València, Spain

## Abstract

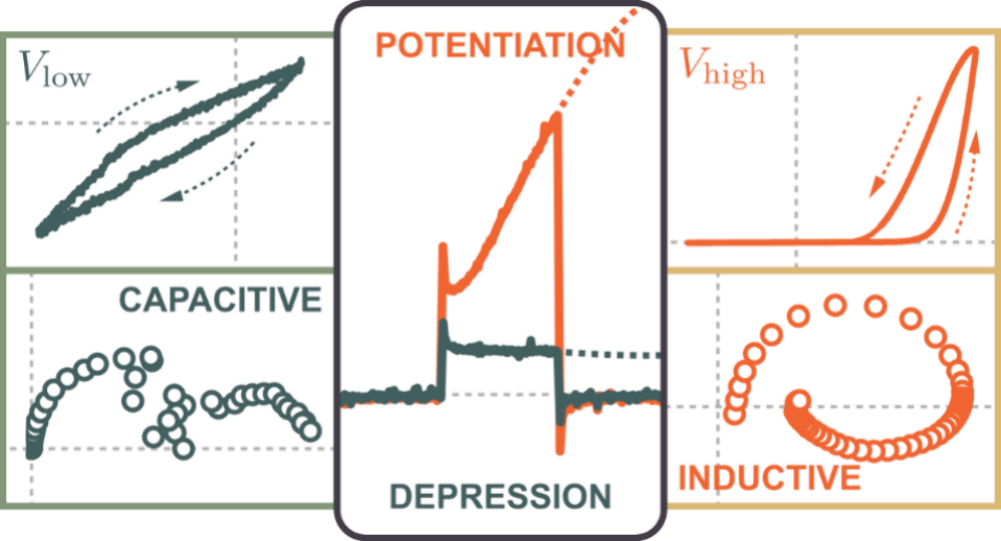

With the increasing demands and complexity of the neuromorphic computing schemes utilizing highly efficient analog resistive switching devices, understanding the apparent capacitive and inductive effects in device operation is of paramount importance. Here, we present a systematic array of characterization methods that unravel two distinct voltage-dependent regimes demonstrating the complex interplay between the dynamic capacitive and inductive effects in volatile perovskite-based memristors: (1) a low voltage capacitance-dominant and (2) an inductance-dominant regime evidenced by the highly correlated hysteresis type with nonzero crossing, the impedance responses, and the transient current characteristics. These dynamic capacitance- and inductance-dominant regimes provide fundamental insight into the resistive switching of memristors governing the synaptic depression and potentiation functions, respectively. More importantly, the pulse width-dependent and long-term transient current measurements further demonstrate a dynamic transition from a fast capacitive to a slow inductive response, allowing for the tailored stimulus programming of memristor devices to mimic synaptic functionality.

## Introduction

Hardware implementation of artificially intelligent devices in bioinspired computing has been gaining significant attention, due to the increasing computation demands of neural network configurations based on traditional von Neumann architecture.^1−3^ Among the novel emerging technologies, resistive random access memories (ReRAMs), also known as memristors, have been widely considered as one of the most promising candidates that can emulate biological neural functions by device physics.^4−6^ Memristors can retain information as the device conductivity, which can be dynamically reconfigured when stimulated by electrical inputs. This unique nature of having both the memory and the processing unit colocated in the same device establishes memristors to be ideally suitable in realizing highly efficient bioinspired neural networks in hardware.^7,8^

The distinctive property of ideal memristive devices is the pinched hysteresis loop with a zero crossing point in their current–voltage (*I*–*V*) characteristic curves arising from the nonlinear dynamics when subjected to a periodic input.^9−11^ However, experimental results from a wide range of memristor device configurations exhibit a nonzero crossing pinched hysteresis loop, which have been attributed to capacitive and inductive contributions.^12−15^ The capacitive effect is observed from the nonzero crossing point,^16−18^ while the inductive effect is observed from the inverse hysteresis loop,^19−21^ typically observed in perovskite-based devices. Notably, these capacitive and inductive characteristics in the *I*–*V* curves of memristor devices have been correlated with the observed impedance spectroscopy measurements,^21,22^ and transient current responses.^23,24^ This indicates that the nonlinear dynamics of the resistive switching is more appropriately related as a change in the overall device impedance.^25^ This complex nonlinear interplay among the resistive, capacitive, and inductive effects is manifested as a frequency-dependent impedance modulation resulting in distinct resistive switching specific to the device configuration.^26^

**Figure 1:**
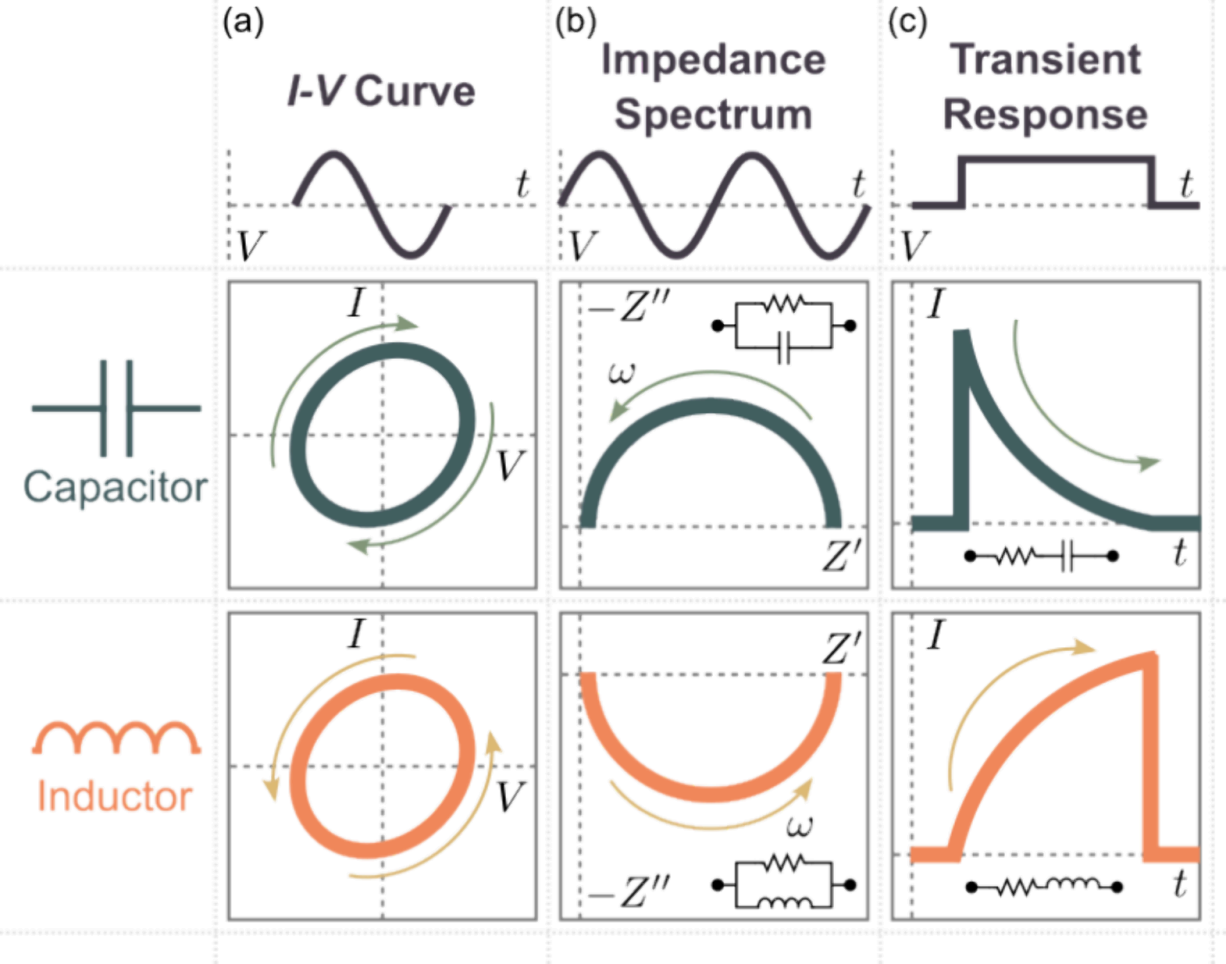
(a) Current–voltage (*I*–*V*) curves of an ideal capacitor and an ideal inductor exhibiting normal and inverted hysteresis, respectively. (b) The impedance spectra of an ideal capacitor and an ideal inductor connected in parallel with a resistor exhibiting a semicircular arc in the first quadrant and the fourth quadrant of the complex plane impedance plot, respectively. (c) The transient current responses of an ideal capacitor and an ideal inductor connected in series with a resistor exhibiting a current decay and an exponential current increase, respectively. The applied voltage stimuli for each characteristic response are indicated by the schematic diagrams.

From classical circuit theory, the current through an ideal capacitor with a capacitance *C* is given by *I* = *C* d*V*/d*t*, while the voltage through an ideal inductor with an inductance *L* is given by *V* = *L* d*I*/d*t*. Upon the application of a periodic voltage input, the *I*–*V* curves (Figure 1a) of the capacitor and the inductor exhibit normal hysteresis (higher current levels in the forward scan than in the reverse scan) and inverse hysteresis (higher current levels in the reverse scan than in the forward scan), respectively. On the other hand, the impedance of the capacitor at an angular frequency ω is given by *Z* = (*i*ω*C*)^−1^, while the inductor is given by *Z* = *i*ω*L*. With the application of a sinusoidal perturbation with varying ω, the impedance spectra (Figure 1b) of a resistor connected in parallel with an ideal capacitor and an ideal inductor exhibit a semicircular arc in the first quadrant and the fourth quadrant of the complex plane impedance plot, respectively. Furthermore, when connected in series with a resistor with a resistance *R*, the transient current response of the capacitor is given by
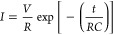
while the inductor is given by
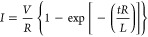
Upon the application of a voltage pulse, the transient current response (Figure 1c) of the capacitor exhibits an exponential decay, while the inductor exhibits an exponential rise. Notably, these capacitive and inductive features have been observed in memristor devices.^13^ Numerous numerical models have been proposed in order to provide a deeper understanding of the underlying mechanisms governing the resistive switching of memristor devices.^11,25,27−29^ However, most of the proposed models describe only the pertinent parameters of the resistive switching of the characteristic *I*–*V* response to simulate neural network algorithms. More importantly, these models do not sufficiently account for the corresponding impedance and transient response of the memristive response providing valuable information on the physical and chemical processes governing switching mechanisms.

Here, we present the extensive characterization of methylammonium lead bromide (MAPbBr_3_) memristors demonstrating the intimate correlation among the characteristic *I*–*V* response, voltage-dependent IS spectral evolution, and the transient current response with both the capacitive and inductive responses. The MAPbBr_3_ perovskite formulation is chosen in order to have a simple and well-established understanding of the mixed electronic and ionic dynamics with low activation energy and high operational stability. Two voltage-dependent regimes are demonstrated: (1) a low-voltage capacitive regime associated with synaptic depression and (2) a high-voltage inductive regime associated with synaptic potentiation. Furthermore, transient measurements with varying pulse durations further demonstrate the interplay between the fast capacitive response and the slow inductive response as observed in the highly correlated IS and current transients. These results provide a more complete picture of the dynamic resistive switching of memristor devices crucial for the development and integration of these artificially intelligent hardware with more complex novel analog neural network algorithms.

**Figure 2:**
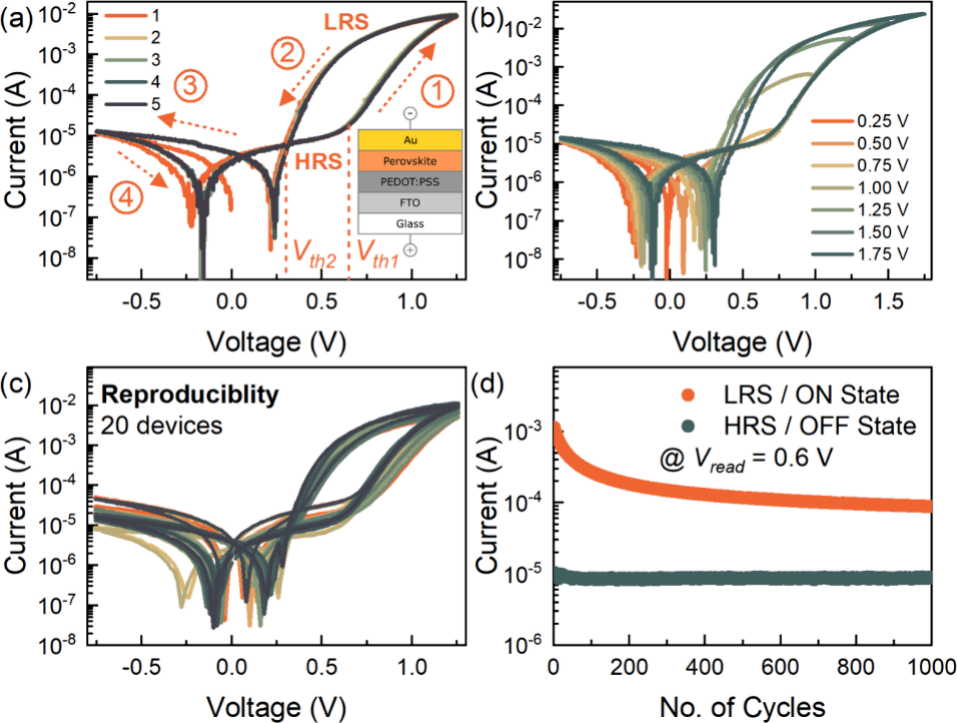
(a) Characteristic *I*–*V* response for 5 cycles at a scan rate of 1 V s^–1^ in the semilogarithmic scale of the FTO/PEDOT:PSS/MAPbBr_3_/Au memristor device exhibiting a threshold resistive switching with the arrows and numbers indicating the scan direction and the inset illustrating the schematic diagram of the device configuration, (b) the upper vertex-dependent multilevel/multistate analog resistive switching of the memristor device, (c) the characteristic *I*–*V* response of 20 distinct devices exhibiting highly reproducible threshold switching, and (d) the endurance measurements for 1000 cycles of the LRS (ON state) and HRS (OFF state) at a read voltage (*V*_read_) of 0.6 V.

The characteristic *I*–*V* response of the perovskite memristor, measured at a scan rate of 1 V s^–1^, on the semilogarithmic scale, is shown in Figure 2a with the inset illustrating the device and measurement configuration. The perovskite memristor exhibits a gradual threshold resistive switching in the positive polarity with an ON/OFF ratio of ∼2 orders of magnitude.^30−32^ At low applied voltages, the device is at its initial high resistance state (HRS) or the OFF state. As the positive voltage scan reaches and goes beyond the initial threshold voltage *V*_th1_ ≈ 0.65 V, the device current gradually transitions from the HRS to the low resistance state (LRS) or ON state promoting the SET process.^33^ On the other hand, in the reverse scan direction, the ON state is maintained until the applied voltage reaches a lower threshold voltage *V*_th2_ ≈ 0.3 V, where the device current transitions from the LRS back to the HRS, promoting the RESET process. The OFF state is then maintained throughout the negative polarity scan. This characteristic resistive switching is considered to have a volatile memory where the ON state relaxes back to the OFF state upon the removal or sufficient reduction of the applied voltage.^5,34,35^ Moreover, by varying the upper vertex voltage of the *I*–*V* measurements (Figure 2b), the device displays a multilevel/multistate resistive switching suitable for analog volatile memory applications in neuromorphic systems.^36−41^ The memristive response of 20 devices is shown in Figure 2c, indicating the robustness and reproducibility of the gradual threshold resistive switching of the device configuration. Endurance measurements of a representative device via cycling for 1000 times demonstrates excellent device operational stability with a sustained ON/OFF ratio of ≥1 order of magnitude at a read voltage of *V*_read_ = 0.6 V (Figure 2d).

**Figure 3:**
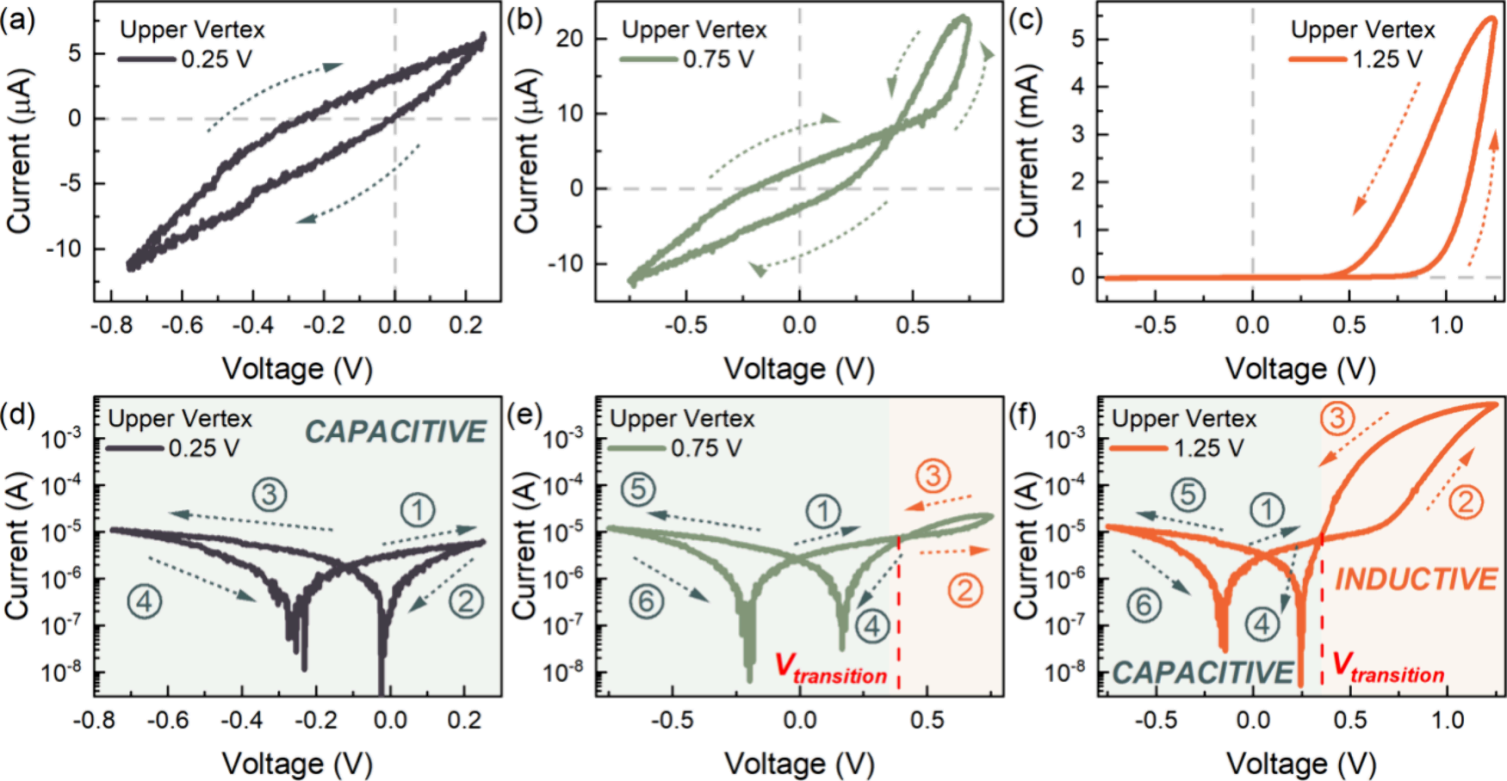
Characteristic *I*–*V* response in the linear scale of the FTO/PEDOT:PSS/MAPbBr_3_/Au memristor device with varying upper vertex voltages of (a) 0.25, (b) 0.75, and (c), 1.25 V, with the arrows indicating the scan direction. Corresponding *I*–*V* response in the semilogarithmic scale for upper vertex voltages of (d) 0.25, (e) 0.75, and (f) 1.25 V, with the arrows and numbers indicating the scan direction and sequence, respectively. [Reproduced with permission from Bisquert, J. Inductive and capacitive hysteresis of current–voltage curves. A unified structural dynamics in solar energy devices, memristors, ionic transistors and bioelectronics, *PRX Energy***2023**, *3*, 011001, licensed under a Creative Commons Attribution (CC BY 4.0) license.]

A closer look at the individual characteristic response of the upper vertex-dependent *I*–*V* curves exhibiting the multistate/multilevel analog memory of the device reveals a transition from a normal to an inverted hysteresis. In the linear scale with an upper vertex of 0.25 V (Figure 3a), the characteristic *I*–*V* response exhibits high current levels in the forward scan direction and low current levels in the reverse scan direction, commonly referred as normal hysteresis, attributed to a fully capacitive response.^13,21,42^ The corresponding characteristic *I*–*V* response in the semilogarithmic scale is shown in Figure 3d, indicating the voltage range in the fully capacitive regime. Increasing the upper vertex up to 0.75 V (Figure 3b), the *I*–*V* response exhibits a pinched hysteresis with a crossing point at ∼0.38 V. This crossing point varies depending on the scan rate of the *I*–*V* measurement, as well as the device operational stability (Figure S1), indicating a dynamic response of the state transitions from capacitive to inductive regimes.^26,43^ Notably, the device consistently exhibits a normal hysteresis for voltages below the crossing point, which transitions to an inverted hysteresis for voltages above the crossing point. This inverted hysteresis loop in the *I*–*V* response is attributed to an inductive time domain response where the forward scan has lower current levels than the reverse scan, typically observed in MAPbBr_3_-based solar cells.^13,21,42^ The corresponding response in the semilogarithmic scale is shown in Figure 3e, indicating the transition from a fully capacitive to an inductive regime. At an even higher upper vertex of 1.25 V (Figure 3c), the device exhibits a predominantly inverted hysteresis, due to the higher current levels. However, from the corresponding semilogarithmic *I*–*V* response (Figure 3f), the fully capacitive region still persists at voltages below the transition voltage, while the strong inductive region is observed at voltages above the transition voltage.

**Figure 4:**
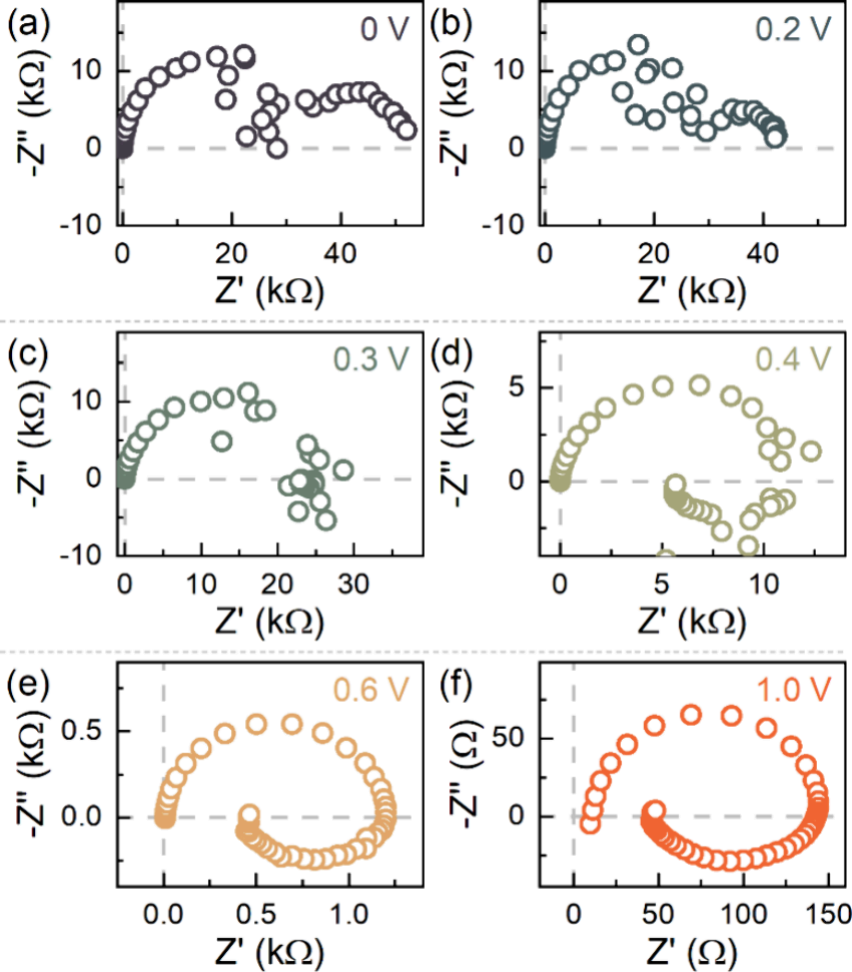
IS spectral evolution at representative applied voltages (*V*_app_) under dark controlled conditions, exhibiting (a, b) a fully capacitive regime at low voltages (*V*_app_< 0.3 V), (c, d) a transition region at intermediate voltages (0.3 V ≤ *V*_app_ ≤ 0.4 V), and (e, f) a low-frequency inductive regime at high voltages (*V*_app_ > 0.4 V).

This inductive response, manifested as the inverted hysteresis, in the time domain *I*–*V* curves, is further corroborated in the frequency domain by tracking the voltage-dependent impedance spectral (IS) evolution. The IS spectral evolution of the perovskite memristor is shown in Figure 4. Notably, the implemented IS measurement protocol corresponds to a very slow *I*–*V* scan close to the steady state.^21,42,44^ At low voltages (*V*_app_ < 0.3 V), the device in the OFF state exhibits a fully capacitive IS response (Figures 4a and 4b). As the voltage approaches 0.3 V (Figure 4c), the low-frequency (LF) capacitive arc begins to decrease and eventually transforms to an LF inductive arc at *V*_app_ = 0.4 V (Figure 4d). Noticeably, a midfrequency noise is observed, which can be attributed to the device state instability, due to the voltage-dependent ion migration dynamics of the mobile Br^–^ species.^45^ At these transition voltages, the current gradually starts to increase, indicating a reduction in the overall device resistance.^32,44^ Beyond these threshold voltages (*V*_app_ ≥ 0.4 V), the high-frequency (HF) capacitance continues to decrease while the LF inductive response is sustained at higher voltages until the device completely switches to the ON state (Figures 4e and 4f). This LF inductive response of the memristor device is not electromagnetic in nature but rather due to the chemical inductor.^24,46−48^

**Figure 5:**
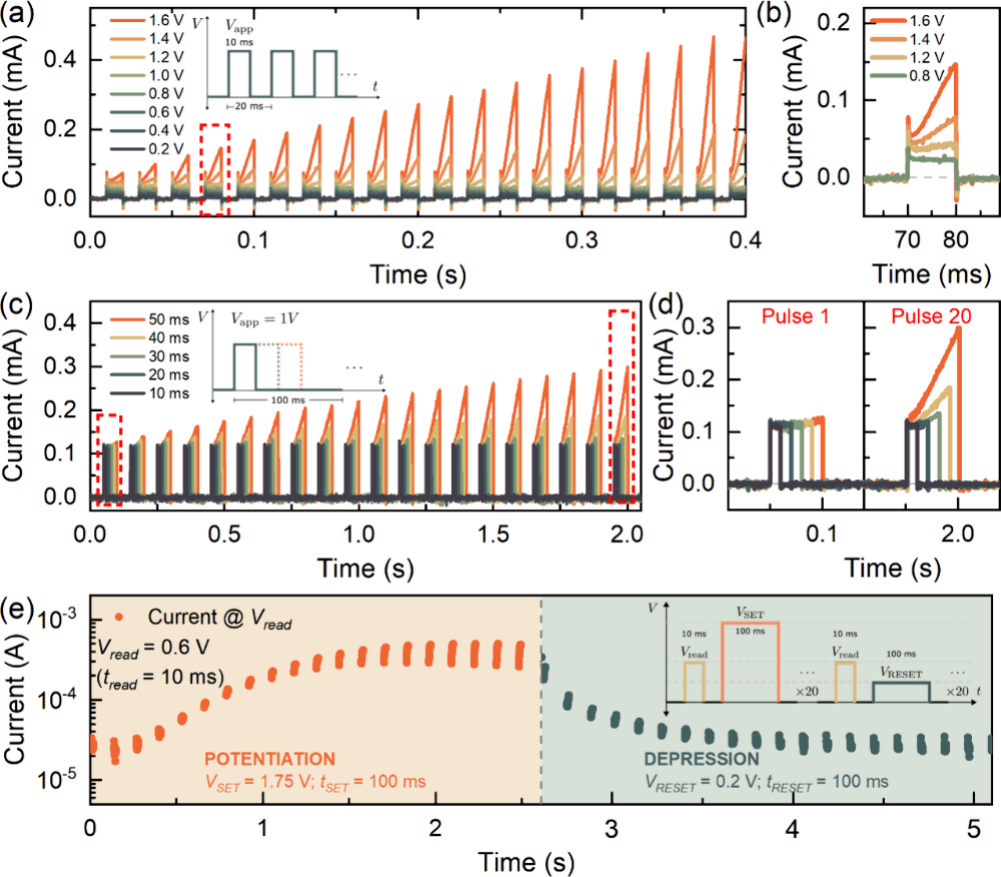
(a) Voltage-dependent transient current response of the perovskite memristor by applying 20 identical voltage pulses with an amplitude of *V*_app_ and a pulse width of 10 ms (schematic diagram shown in inset), (b) the magnified view of the transient current response of a single voltage pulse at representative *V*_app_ levels, (c) the pulse width-dependent transient current response by applying 20 identical voltages pulses with a period *T*_pulse_ of 100 ms, (d) the corresponding magnified view of the first and last transient responses, and (e) the synaptic potentiation and depression characteristic response of the memristor measured at a read voltage *V*_read_ = 0.6 V (pulse width *t*_read_ = 10 ms), SET voltage *V*_SET_ = 1.75 V (pulse width = 100 ms), and a RESET voltage *V*_RESET_ = 0.2 V (pulse width = 100 ms) with the inset illustrating the schematic diagram of the pulsed measurement sequence.

Not only is the inductive response observed in the characteristic *I*–*V* (strong inverted hysteresis) and IS (LF inductive arc), but it is also observed in the transient current response. The voltage-dependent transient response of the perovskite memristor using a train of 20 identical voltage pulses with a pulse width (*t*_pulse_) of 10 ms and a period (*T*_pulse_) of 20 ms is shown in Figure 5a. At low applied voltages (*V*_app_ < 1 V), the current level is maintained throughout the full voltage train. However, at higher applied voltages (*V*_app_ ≥ 1 V), the current begins to gradually increase with every succeeding voltage pulse. This gradual increase in current response with the subsequent application of voltage pulses is the distinctive phenomenon of synaptic potentiation.^41,49,50^ A closer look at the transient response of a single voltage pulse (Figure 5b) at applied voltages lower than the threshold voltage, a sharp initial transient peak is observed, subsequently followed by a gradual decay attributed to a capacitive charging of the device.^24,51^ In contrast, at higher applied voltages, a gradual increase in current is observed after the gradual decay, indicating the slow inductive contribution once the capacitor has been charged, further reducing the total resistance.^51^ Finally, a sharp negative current peak is observed upon the removal of the voltage pulse due to the internal voltage and the series resistance of the device.^51,52^ This correlation of the impedance characteristics and transient behavior has been well-established by neuron-style models in halide perovskite solar cells and memory devices.^53−55^ Moreover, this transient response of the voltage-dependent potentiation has been observed in voltage-gated potassium channels abundantly expressed in the human brain.^56^

In addition to the voltage-dependent transient current response of the memristor device, the synaptic potentiation behavior can also be realized via pulse width-dependent measurements shown in Figure 5c. With a *V*_app_ = 1 V and a pulse period of 100 ms, the voltage trains with short pulse widths of ≤30 ms exhibit no potentiation after 20 pulses (Figure 5d). This indicates that the interval between the two pulses is long enough that the device relaxes back to the initial HRS before the next pulse arrives.^50^ On the other hand, the longer pulse widths of >30 ms demonstrate the inductive effect of the gradual current increase leading to the synaptic potentiation of the device upon the application of 20 identical pulses. This potentiation due to the pulse width implies that the memory effect is closely related to the slow time scales of the voltage-dependent inductive response being retained with the subsequent application of voltage pulses.

From the voltage- and pulse width-dependent transient current response of the memristor device, the synaptic potentiation and depression of the resistance state via a read voltage (*V*_read_) for operational applications is demonstrated as shown in Figure 5e.^57,58^ By applying a *V*_read_ < *V*_th1_ with a short pulse width of *t*_read_ = 10 ms and a relatively higher SET voltage *V*_SET_ = 1.75 V with a longer pulse width of *t*_SET_ = 100 ms, the memristor potentiates from the OFF state to the ON state after the subsequent application of 20 identical voltage pulses. The short *t*_read_ ensures that the device does not exhibit potentiation at low *V*_read_ values while the longer *t*_SET_ allows the memristor to probe its inductive response. In contrast, by applying a lower positive RESET voltage *V*_RESET_ = 0.2 V with *t*_RESET_ = 100 ms, the device exhibits synaptic depression, making the device transition from the ON state back to the OFF state. This sufficiently low but still positive *V*_RESET_ further confirms the volatile memory characteristics of the perovskite memristor device. The full transient response of the device at representative SET and RESET processes during the synaptic potentiation and depression measurements, respectively, is shown in Figure S2.

**Figure 6:**
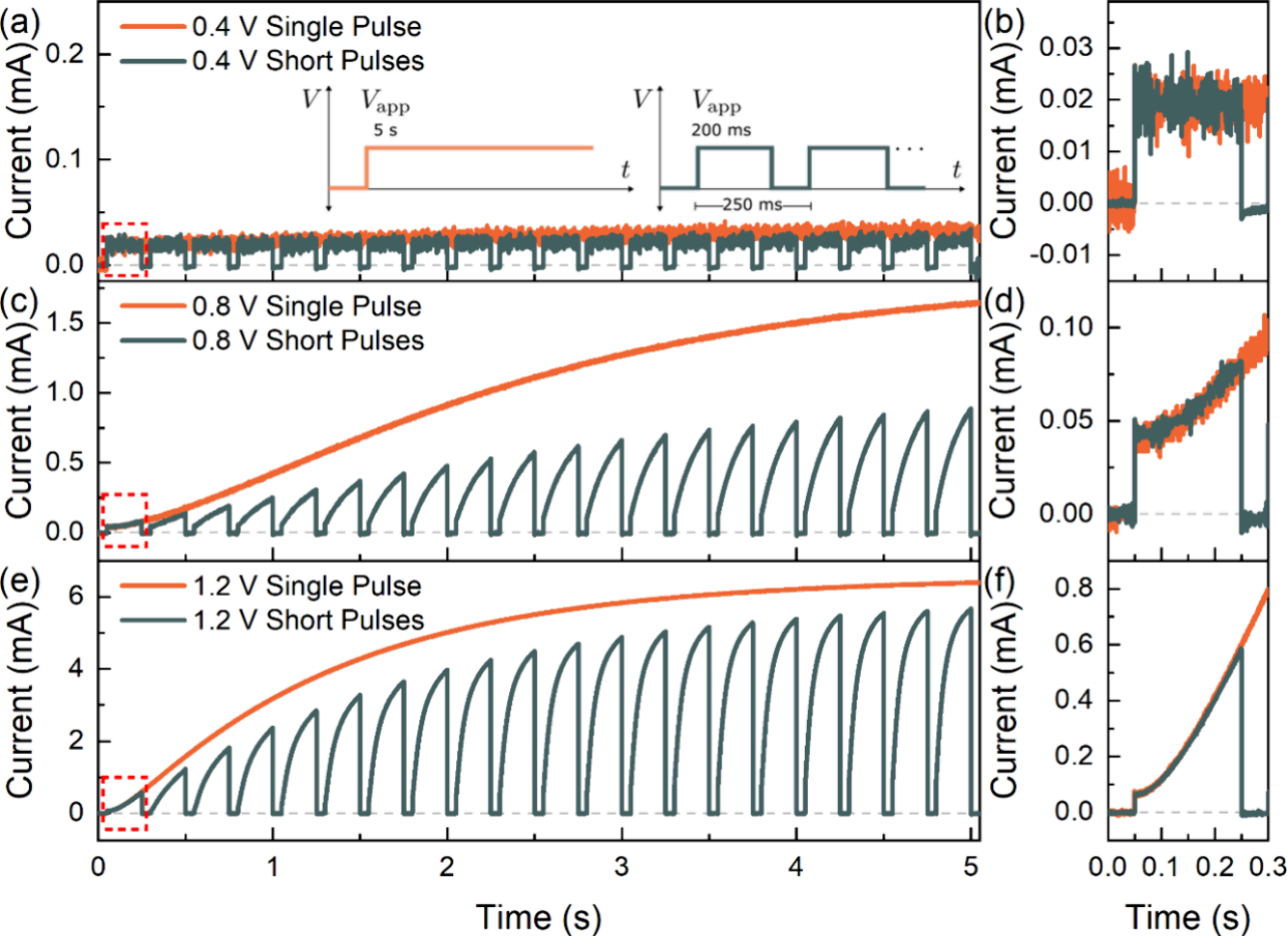
Transient current response of the volatile perovskite memristor with a single long pulse (5 s) and a train of 20 identical short pulses (pulse width of 200 ms; pulse period of 250 ms) at applied voltages of (a) 0.4 V, (c) 0.8 V, and (e) 1.2 V with the inset showing the schematic diagram of the applied voltage stimuli. Panels (b), (d), and (f) correspond to the magnified view of the first voltage pulse of panels (a), (c), and (e), respectively.

As the synaptic potentiation is demonstrated to be intimately correlated with the adequate voltage applied and response time of the inductive effect of the memristor, the long-term transient current profile provides essential information on the suitable conditions for promoting this potentiation.^24^ The comparison of the voltage-dependent transient current response of the perovskite memristor between a single long voltage pulse (5 s) and a voltage train of 20 shorter pulses (pulse width of 200 ms and pulse period of 250 ms) is shown in Figure 6, in which three different domains can be distinguished.

(i) For an applied voltage lower than the SET threshold voltage *T*_th1_ (Figure 6a), the long-term transient profile exhibits a small initial current peak, followed by a slight current decay with no potentiation and the device stays in the OFF state (Figure 6b). This indicates that, at this applied potential, the response is fully capacitive and the inductive effect of the memristor is not promoted, even for longer durations.^51^ Consequently, for the short pulses, no synaptic potentiation is observed.
(ii) For an applied voltage above *V*_th1_ (Figure 6c), the small initial peak with a slight current decay is again observed, but this time, the current gradually increases, switching the device to the ON state. This potentiation of the single long pulse implies that, at applied voltages higher than *V*_th1_, the inductive response of the device is activated further reducing the device resistance, consistent with the LF IS response.^23,44^ Consistently, the first pulse of the voltage train exhibit the same sharp transient peak and current decay; however, the inductive response is activated within the duration of the pulse (Figure 6d) and potentiation is observed with 20 pulses. Notably, the ON state current of the single pulse is not obtained by the voltage train due to the volatile memory of the device, which gradually promotes the short RESET process when the applied voltage is at 0 V between the pulses.
(iii) Finally, for applied voltages further beyond the *V*_th1_ (Figure 6e), the rate of current increase after the initial transient peak is faster, reaching the ON state current within a shorter period. This higher potentiation rate indicates that, at higher applied voltages, the contribution of the inductive effect is more dominant. Correspondingly, the first pulse of the voltage train exhibits the same faster current increase promoting synaptic potentiation with ON state current closer to that of the single long pulse (Figure 6f). This indicates that (a) the inductive contribution is high during the 200 ms pulse width and (b) the 50 ms duration at 0 V only promotes a slight synaptic depression, resulting in current levels closer to that of the long single voltage pulse. The higher ON state current levels of the pulsed stimulus indicate that the duration of the RESET process between the pulses is substantially short, maintaining the device closer to the LRS. The long-term transient current profile of the device response allows for the proper identification of the voltage, pulse width, and period of the applied stimulus, promoting synaptic potentiation, which is crucial to the device implementation in more-complex neural network configurations.

## I

In summary, we have demonstrated the complex interplay of the characteristic capacitive and inductive effects in volatile perovskite memristors via extensive characterization of the electrical and transient responses. Systematic investigation of the voltage-dependent electrical characterizations of the perovskite memristor unravel two distinct regimes: (1) low-voltage capacitance-dominant response evidenced by the normal hysteresis and nonzero crossing in the characteristic –*V* curves, low-frequency capacitive arcs in the IS response, and an exponential current decay in the transient measurements exhibiting synaptic depression; (2) high-voltage inductance-dominant response evidenced by the inverted hysteresis and resistance modulation in the *I*–*V* curves, low-frequency inductive arcs in the IS response, and a gradual current buildup in the transient measurements exhibiting synaptic potentiation. Furthermore, in the inductance-dominant regime, transient measurements further demonstrate the interplay between the dynamic characteristic times of a fast capacitive response, followed by a slower inductive response. This complete set of characteristic features provides an integrated picture of the capacitive and inductive responses, as observed in highly correlated *I*–*V*, IS, and transient current measurements. These responses enable a full understanding of the potentiation and depression of halide perovskite memristors applied as synaptic memory elements. The kinetic time scales of the voltage-dependent characteristic capacitive and inductive responses of memristors facilitate the development of highly efficient, versatile neural network algorithms utilizing these artificially intelligent devices.

## Supporting Information Available

Detailed experimental details of the MAPbBr_3_ perovskite memristor device fabrication, electrical characterization, impedance spectral evolution measurement protocol, voltage- and pulse width-dependent transient current response, and synaptic potentiation and depression measurements. Additional information on the scan rate-dependent characteristic *I*–*V* response and endurance measurements, with the full transient response of the synaptic potentiation and depression measurements (PDF)
