# Supplementary material for: Capacitive and Inductive Characteristics of Volatile Perovskite Resistive Switching Devices with Analog Memory
Source: J Phys Chem Lett. 2024 Jun 13;15(25):6496–503. doi: 10.1021/acs.jpclett.4c00945 (PMC11215770; doi:10.1021/acs.jpclett.4c00945)
Supplement: Supplementary file 1 — jz4c00945_si_001.pdf [file jz4c00945_si_001.pdf]

Supporting Information

Capacitive and inductive characteristics of volatile perovskite resistive switching devices with analog memory

Cedric Gonzales*,[1] Agustín Bou,[1,2] Antonio Guerrero,[1] Juan Bisquert[1,2]

[1]Institute of Advanced Materials (INAM), Universitat Jaume I, 12006 Castelló, Spain.
[2]Leibniz-Institute for Solid State and Materials Research Dresden, Helmholtzstraße 20, 01069 Dresden, Germany
[3]Instituto de Tecnología Química (Universitat Politècnica de València-Agencia Estatal Consejo Superior de Investigaciones Científicas), Av. dels Tarongers, 46022, València, Spain.

Corresponding author: (gonzalek@uji.es)

Experimental Details

Device Fabrication

The fluorine-doped tin oxide (FTO) substrates (TEC15) were partially etched with zinc powder and a 2 M hydrochloric acid solution. The etched samples were individually brushed to mechanically remove the residues of the etching process. The brushed samples were then subjected to a sequence of 15-minute sonication in deionized water with Hellmanex detergent solution, acetone, and isopropyl alcohol. The cleaned samples were blow-dried using a nitrogen gun.

Prior to the deposition of the poly(3,4-ethylenedioxythiopene) polystyrene sulfonate (PEDOT:PSS), the cleaned samples were subjected to an ultraviolet-ozone (UV-O_3) treatment for 15 minutes to further remove organic contamination on the surface and improve the surface wetting. The PEDOT:PSS solution (Clevios P VP. Al 4083) was filtered using a 0.45 µm Nylon filter and was statically spin coated onto the etched FTO substrates for 30 s at 3000 RPM with an acceleration of 1000 RPM/s. The deposited PEDOT:PSS was annealed at 100 °C for 5 minutes then was immediately transported into a nitrogen-controlled glove box in preparation for the $MAPbBr_3$ perovskite deposition.

A 1.4 M $MAPbBr_3$ precursor solution was prepared using $PbBr_2$ (>98%, TCI), and MABr (>99.99%, Greatcell Solar) in 1:4 dimethylsulfoxide (DMSO) (≥99.9%, Sigma Aldrich) : N,N-dimethylformamide (DMF) (99.8%, Sigma Aldrich) solution. A 50 µL $MAPbBr_3$ perovskite solution was statically spin coated onto the PEDOT:PSS layer via a two-step antisolvent method: 10 s at 1000 RPM, followed by 40 s at 4000 RPM. A 100 µL toluene (99.8%, Sigma Aldrich) antisolvent was injected 32 s before the spin coating ended. The samples were then annealed at 100 °C for 30 minutes.

Finally, an 85 nm gold contact was thermally evaporated using a commercial Oerlikon Leybold Univex 250.

Electrical Characterization

All electrical characterization measurements were measured inside a nitrogen-controlled glove box under dark conditions using an Autolab PGSTAT204. The characteristic $I - V$ curves of the perovskite devices were measured with varying scan rates and upper vertex voltages. The reproducibility and endurance measurements of the 20 devices were measured at a scan rate of 1 V s^{-1}.

Impedance Spectral Evolution Measurement Protocol

The dynamic transition state was investigated via a sequence of chronoamperometry (CA) for 5 s subsequently followed by impedance spectroscopy (IS) with a frequency range of 0.1MHz – 0.02 Hz and an amplitude of 10 mV. The CA-IS sequence was conducted at varying voltages from 0 V to 1.3 V back to 0V at 0.1 V increments.

Voltage-dependent Transient Current Response

The voltage-dependent transient current response of the device at an applied voltage (V_{app}) pulse train were measured using 20 voltage pulses with a pulse width (t_{pulse}) of 10 ms and a pulse period (T_{pulse}) of 20 ms. The V_{app} was varied from 0.2 V to 1.6 V at a voltage interval of 0.1 V.

Pulse Width-dependent Transient Current Response

The pulse width-dependent transient current responses of the device were measured at V_{app} = 1 V using 20 voltage pulses with varying t_{pulse} of 10 – 50 ms and a T_{pulse} of 100 ms. The device was RESET back to the OFF state prior to every measurement.

Potentiation and Depression Measurements

The synaptic potentiation and depression characteristic response of the memristor measured at a read voltage V_{read} = 0.6 V (pulse width t_{read} = 10 ms), SET voltage V_{SET} = 1.75 V (pulse width = 100 ms), and a RESET voltage V_{RESET} = 0.2 V (pulse width = 100 ms)

Scan Rate-Depedent Characteristic $I - V$ and Endurance Measurements

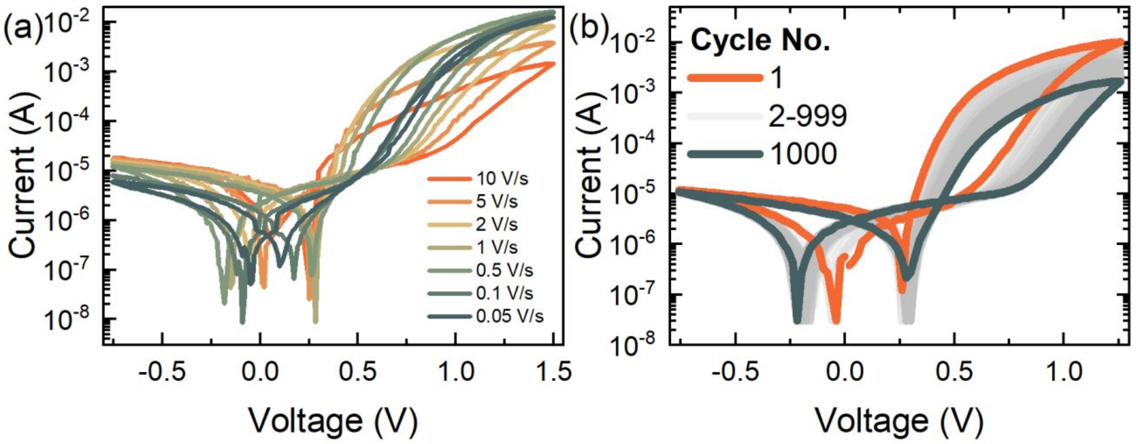

Figure S1. (a) The scan rate-dependent characteristic $I - V$ curves of the perovskite memristor and (b) the endurance measurements for 1000 cycles both exhibiting the varying crossing point of the transition from a capacitive to an inductive hysteresis.

Full Transient Current Response of the Synaptic Potentiation and Depression

The full transient response of the device at representative SET and RESET processes during the synaptic potentiation and depression measurements, respectively, is shown in Fig. S2. Initially, at the first V_{read} = 0.6 V, the current response exhibits an initial OFF state of ~25 μA (Fig. S2b). With the application of the first SET pulse of V_{SET} = 1.75 V, the inductive gradual current increase is observed consistent with voltages beyond the threshold voltage. The consecutive application of SET voltage pulses gradually increases the current response at the same V_{read} approaching the ON state (~135 μA) promoting the synaptic potentiation (Fig. S2c). On the other hand, the application of a second RESET pulse of V_{RESET} = 0.2 V to the device already in the ON state exhibits capacitive decay which gradually lowers the current levels of the subsequent V_{read} device state (Fig. S2d). In contrast, the consecutive RESET voltages pulses gradually decrease the current response at V_{read} from the ON state current back to the initial OFF state current promoting the synaptic depression (Fig. S2e). Notably, at the steps where the applied voltage is 0 V, the negative transient spike and decay is consistently observed for both the synaptic potentiation and depression measurements consistent with the capacitive discharge phase of the device.

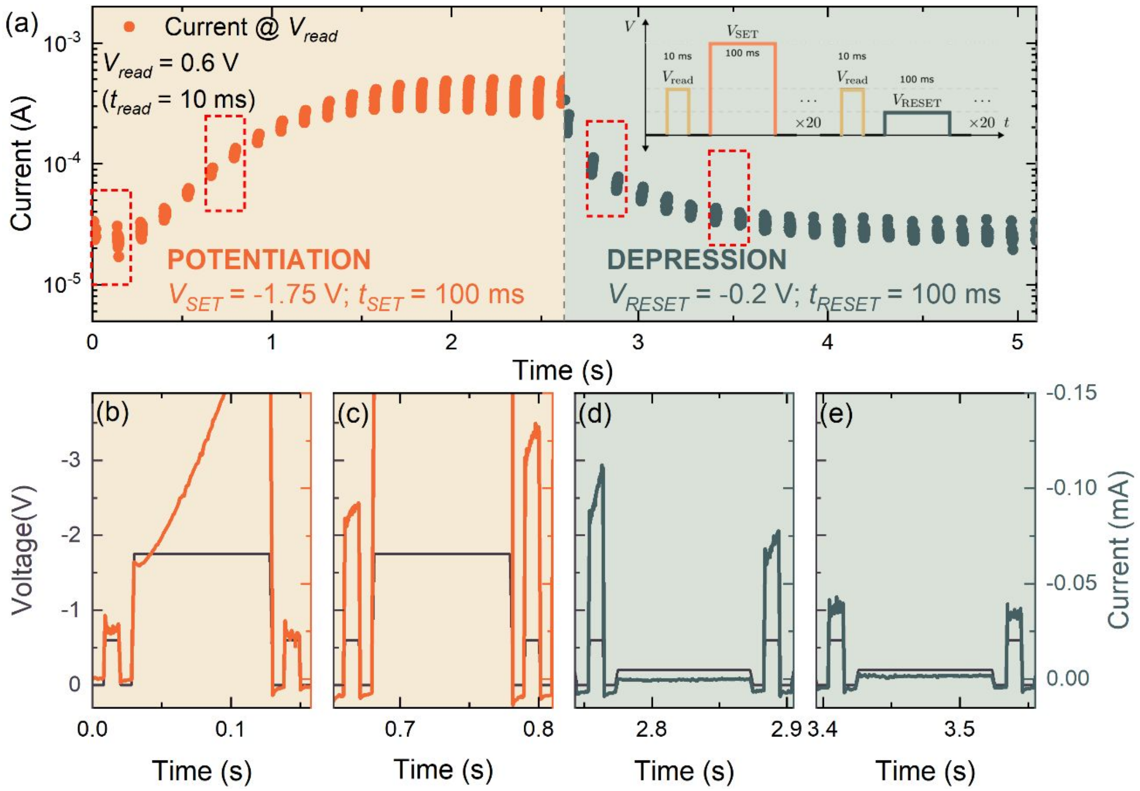

Figure S2. (a) The synaptic potentiation and depression characteristic response of the memristor measured at a read voltage V_{read} = 0.6 V (pulse width t_{read} = 10 ms), SET voltage V_{SET} = 1.75 V (pulse width = 100 ms), and a RESET voltage V_{RESET} = 0.2 V (pulse width = 100 ms) with the inset illustrating the schematic diagram of the pulsed measurement sequence, the magnified view of the full transient response during synaptic potentiation of the a pair of succeeding V_{read} states after (b) the first and (c) sixth V_{SET} pulses, and the magnified view of the full transient response during synaptic depression of the a pair of succeeding V_{read} states after (b) the second and (c) seventh V_{RESET} pulses.

This synaptic depression of memristors have been recently investigated as the "conduction capacitor" which accompanies the chemical inductor.[1] Moreover, a general form of the model shows the transient response of the synaptic depression wherein the current decreases when the device is at its capacitive-dominant regime.[2, 3] Finally, an experimental demonstration of the synaptic depression due to the capacitive conductance has been reported recently for nanofluidic pores exhibiting memristive response.[4]

References

(1) Bisquert, J.; Roldán, J. B.; Miranda, E. Hysteresis in memristors produces conduction inductance and conduction capacitance effects. *Physical Chemistry Chemical Physics* **2024**, *26* (18), 13804-13813. DOI: 10.1039/D4CP00586D.

(2) Hernández-Balaguera, E.; Bisquert, J. Negative Transient Spikes in Halide Perovskites. *ACS Energy Letters* **2022**, *7* (8), 2602-2610. DOI: 10.1021/acsenergylett.2c01252.

(3) Bisquert, J. Hysteresis, Impedance, and Transients Effects in Halide Perovskite Solar Cells and Memory Devices Analysis by Neuron-Style Models. *Advanced Energy Materials* **2024**, *2400442*. DOI: 10.1002/aenm.202400442.

(4) Ramirez, P.; Portillo, S.; Cervera, J.; Bisquert, J.; Mafe, S. Memristive arrangements of nanofluidic pores. *Physical Review E* **2024**, *109* (4), 044803-044803. DOI: 10.1103/PhysRevE.109.044803.
